# Supplementary material for: Splice-Junction-Based Mapping of Alternative Isoforms in the Human Proteome
Source: Cell Rep. Author manuscript; Available in PMC 2020 Jan 15. (PMC6961840; doi:10.1016/j.celrep.2019.11.026)
Supplement: 2 [file NIHMS1546469-supplement-2.zip › DF1/PXD006675/VentricularSeptum/VentricularSeptum_26_TTN_ASHTEEEVSVTVPEVPK.pdf]

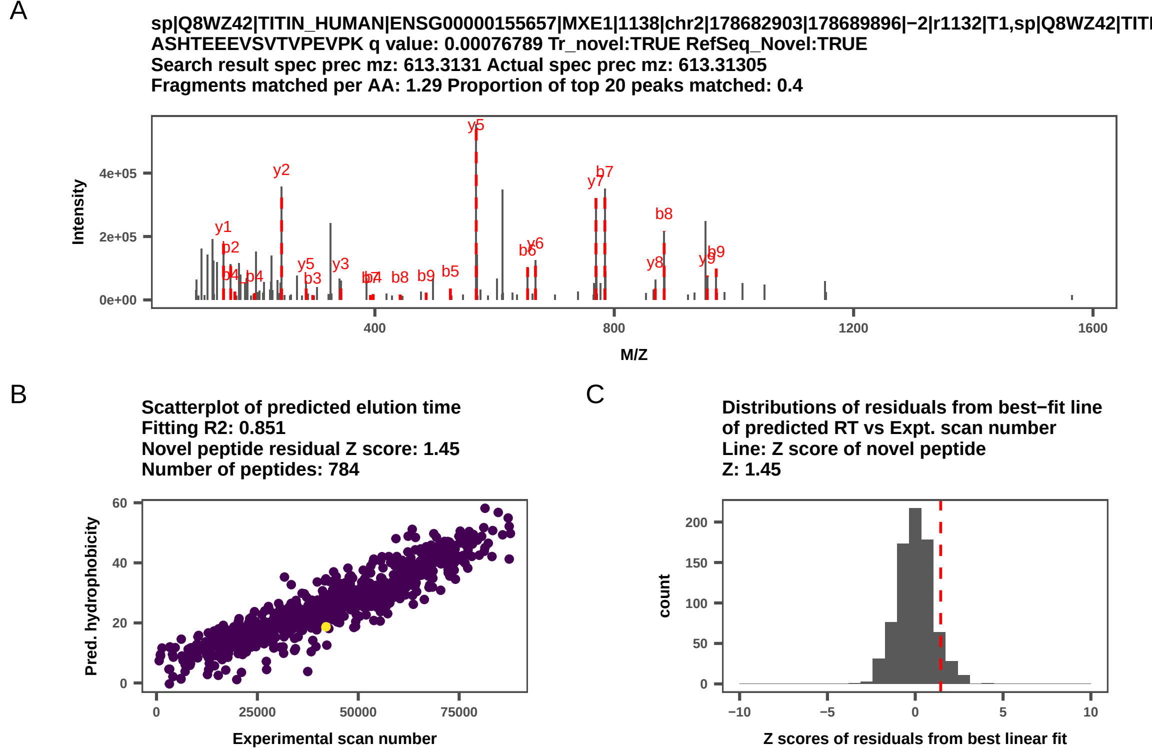
A
sp|Q8WZ42|TITIN_HUMAN|ENSG00000155657|MXE1|1138|chr2|178682903|178689896|−2|r1132|T1,sp|Q8WZ42|TITI
ASHTEEEVSVTVPEVPK q value: 0.00076789 Tr_novel:TRUE RefSeq_Novel:TRUE
Search result spec prec mz: 613.3131 Actual spec prec mz: 613.31305
Fragments matched per AA: 1.29 Proportion of top 20 peaks matched: 0.4
Intensity
4e+05
2e+05
0e+00
y5
y2
b7
y7
b8
y1
b2
y6
b6
y5
y3
y8
b9
y9
b4
b4
b3
b7
b4
b8
b9
b5
400
800
1200
1600
M/Z
B
Scatterplot of predicted elution time
Fitting R2: 0.851
Novel peptide residual Z score: 1.45
Number of peptides: 784
Pred. hydrophobicity
60
40
20
0
0
25000
50000
75000
Experimental scan number
C
Distributions of residuals from best−fit line
of predicted RT vs Expt. scan number
Line: Z score of novel peptide
Z: 1.45
count
200
150
100
50
0
−10
−5
0
5
10
Z scores of residuals from best linear fit
